# Editorial: Current Challenges and Future Perspectives on Emerging Bioelectrochemical Technologies

**DOI:** 10.3389/fmicb.2016.00860

**Published:** 2016-06-08

**Authors:** Tian Zhang, Pier-Luc Tremblay

**Affiliations:** ^1^The Novo Nordisk Foundation Center for Biosustainability, Technical University of DenmarkHørsholm, Denmark; ^2^School of Chemistry, Chemical Engineering and Life Science, Wuhan University of TechnologyWuhan, China

**Keywords:** bioelectrochemical system, extracellular electron transfer, bioelectrode, microbial catalyst, microbial electrosynthesis, microbial fuel cell, bioremediation, *c*-type cytochromes

In its simplest form, bioelectrochemical systems (BESs) consist of an anode, a cathode, and a microbial catalyst (Rabaey and Rozendal, 2010). One of the attractive features of bioelectrochemical technology is that, BESs can be developed to implement anodic-based or cathodic-based bioprocesses or both at the same time. For example, pollutants can be degraded by a specialized microbial catalyst at the anode while the electrons and protons generated can be used by a biocathode to synthesize useful chemicals.

Among the notable applications of BESs, the most extensively studied is microbial fuel cell, a technology developed over the last century for the production of electrical energy from chemical substrates oxidized by a microbial catalyst at the anode (Potter, 1911; Logan and Rabaey, 2012). BESs can also be used to reduce CO_2_ into methane or multicarbon chemicals including biofuels at the cathode with external electrical energy coming from renewable energy sources (Lovley and Nevin, 2013; Tremblay and Zhang). Besides greenhouse gas emissions control, one of the purposes of this application termed microbial electrosynthesis is to store electricity surplus in ready-to-use chemicals. A derived technology consists in powering a BESs reactor with solar cell for the production of chemicals thus mimicking natural photosynthesis. This approach is intensively pursued because it has the potential to have a solar-to-chemicals conversion efficiency significantly higher than traditional photosynthetic biomass-based bioprocesses (Nevin et al., 2010; Zhang, 2015). In the domain of chemicals production, BESs are also developed to electrify white biotechnologies where the substrates of the microbial catalyst are organic carbon molecules (Choi et al., 2014; Harnisch et al., 2015). In this case, BESs are employed to optimize the redox balance of production bioprocesses resulting in improved yield and profitability.

Additionally, BESs are used for environment-cleaning applications aiming for example at polishing wastewaters or at removing petroleum derivatives, heavy metals, azo dyes, and chlorinated organic compounds from contaminated sites (Logan and Rabaey, 2012; Wang et al., 2015). Employing BESs for bioremediation provides an unceasing source of electron acceptor or donor eliminating the need for costly chemical amendments. Water desalination, biosensors, H_2_ production, and investigative tools to monitor biological activity are other bioelectrochemical technologies of note (Cao et al., 2009; Tremblay and Zhang).

Although BESs are versatile systems with promising features, most bioelectrochemical technologies developed until now have been restricted to lab scale. Low production rate and limited efficiency have stymied scaling up. To overcome this challenge, electroactive microbial catalysts as well as electrochemical reactor components and design must be studied in details for the development of rational optimization strategies. Furthermore, BESs are non-traditional environments for microbes and knowledge on these systems may not be sufficient at the moment for effective optimization. This concerns particularly electron transfer mechanisms between microbes and electrodes which are central to bioelectrochemical technologies and must be well understood.

In this topic, microbe–electrode interface, electron transfer, and associated regulation mechanisms are covered with review articles and original research articles. Kracke et al. discussed in their review electron transfer in BESs performing cathodic-based bioprocesses. This article describes different electron transport hypotheses and their impact on microbial cell redox and energy levels. Kouzuma et al. focused on intracellular catabolic pathways and extracellular electron transfer (EET) in *Shewanella oneidensis*, a model bacterium for the study of EET transferring electrons to solid donors such as anode electrode. In their review, the authors elaborated on the central carbon metabolism, the electron transport chain from the cytoplasm to the final electron acceptor as well as on the involved regulation network. Dantas et al. examined EET in another important model bacterium for electron transfer to solid acceptor, *Geobacter sulfurreducens*. More specifically, the authors focused on the function and structure of multiheme c-type cytochromes in EET by discussing the considerable work done with the model periplasmic triheme c-type cytochrome PpcA. In their original research study, Alves et al. demonstrated the central role of the small tetraheme *c*-type cytochrome STC in periplasmic electron transfer during anaerobic respiration in *S. oneidensis*. Furthermore, this study showed functional redundancy between STC and the flavocytochrome *c* FccA in the electron transport chain responsible for the robustness of this pathway.

Research articles studying microbial ecosystem and physiology with bioelectrochemical tools are also presented in this topic. Ha et al. studied the impact of varying electrochemical conditions on the formation and electron transfer processes between phototrophic microbial mats and electrodes. Ishii et al. demonstrated that the Fe(II)-oxidizing bacterium *Acidithiobacillus ferrooxidans* uses electrical current as source of energy, which is a metabolism possibly involved in carbon assimilation at deep-sea vent system. Friedman et al. showed that the redox environment of a stream riparian zone can be manipulated with a poised electrode as an electron acceptor resulting in lower CH_4_ emissions.

Furthermore, this topic included studies on conception and optimization of novel BES reactors for different applications. Giddings et al. analyzed the performance of a simplified microbial electrosynthesis reactor coupled with a direct power source with no separation membrane between the anode and the cathode. Rodenas Motos et al. developed a novel microbial fuel cell configuration limiting internal voltage losses in a system simultaneously generating electrical current and recovering copper. Cruz Viggi et al. proved the concept of the “oil-spill snorkel,” a bioelectrochemical technology aiming at stimulating the degradation of petroleum contaminants. The “oil-spill snorkel” consists in a single electrode half-buried in sediments where it serves as electron acceptor for the biological oxidation of contaminants. The electrons then flow toward the other half of the electrode exposed in the water serving as a cathode reducing O_2_.

With this topic, we presented examples of applications as well as optimization strategies developed to translate lab scale BES experiments into applicable technologies. The potential of BESs as investigative tools to study microbial physiology and ecology was also shown. Furthermore, we assembled a detailed portrait of the EET mechanisms involved in microbes–electrode interactions. BES technologies are still at an early stage of development and considerable effort must still be expanded by researchers to ensure a fruitful progression toward a bright future.

## Author contributions

TZ and PT co-edited this topic and wrote this editorial.

## Funding

This work was funded by the Novo Nordisk Foundation.

### Conflict of interest statement

The authors declare that the research was conducted in the absence of any commercial or financial relationships that could be construed as a potential conflict of interest.
